# Toward an Integrated Machine Learning Model of a Proteomics Experiment

**DOI:** 10.1021/acs.jproteome.2c00711

**Published:** 2023-02-06

**Authors:** Benjamin A. Neely, Viktoria Dorfer, Lennart Martens, Isabell Bludau, Robbin Bouwmeester, Sven Degroeve, Eric W. Deutsch, Siegfried Gessulat, Lukas Käll, Pawel Palczynski, Samuel H. Payne, Tobias Greisager Rehfeldt, Tobias Schmidt, Veit Schwämmle, Julian Uszkoreit, Juan Antonio Vizcaíno, Mathias Wilhelm, Magnus Palmblad

**Affiliations:** † National Institute of Standards and Technology, Charleston, South Carolina 29412, United States; ‡ Bioinformatics Research Group, University of Applied Sciences Upper Austria, Softwarepark 11, 4232 Hagenberg, Austria; § VIB-UGent Center for Medical Biotechnology, VIB, 9000 Ghent, Belgium; ∥ Department of Proteomics and Signal Transduction, Max Planck Institute of Biochemistry, 82152 Martinsried, Germany; ⊥ Institute for Systems Biology, Seattle, Washington 98109, United States; # MSAID GmbH, 10559 Berlin, Germany; ⊗ Science for Life Laboratory, KTH - Royal Institute of Technology, 171 21 Solna, Sweden; ¶ Department of Biochemistry and Molecular Biology, University of Southern Denmark, 5230 Odense, Denmark; ∇ Department of Biology, Brigham Young University, Provo, Utah 84602, United States; ▲ Institute for Mathematics and Computer Science, University of Southern Denmark, 5230 Odense, Denmark; △ MSAID GmbH, 85748 Garching, Germany; ▼ Medical Proteome Analysis, Center for Protein Diagnostics (ProDi), Ruhr University Bochum, 44801 Bochum, Germany; ◆ European Molecular Biology Laboratory, European Bioinformatics Institute (EMBL-EBI), Wellcome Trust Genome Campus, Hinxton, Cambridge CB10 1SD, United Kingdom; ◇ Computational Mass Spectrometry, Technical University of Munich (TUM), 85354 Freising, Germany; ○ Leiden University Medical Center, Postbus 9600, 2300 RC Leiden, The Netherlands; ● Department of Biomolecular Medicine, Faculty of Health Sciences and Medicine, Ghent University, 9000 Ghent, Belgium; □ Medizinisches Proteom-Center, Medical Faculty, Ruhr University Bochum, 44801 Bochum, Germany

**Keywords:** machine learning, deep learning, artificial intelligence, synthetic data, enzymatic digestion, liquid chromatography, ion mobility, tandem mass spectrometry, research integrity

## Abstract

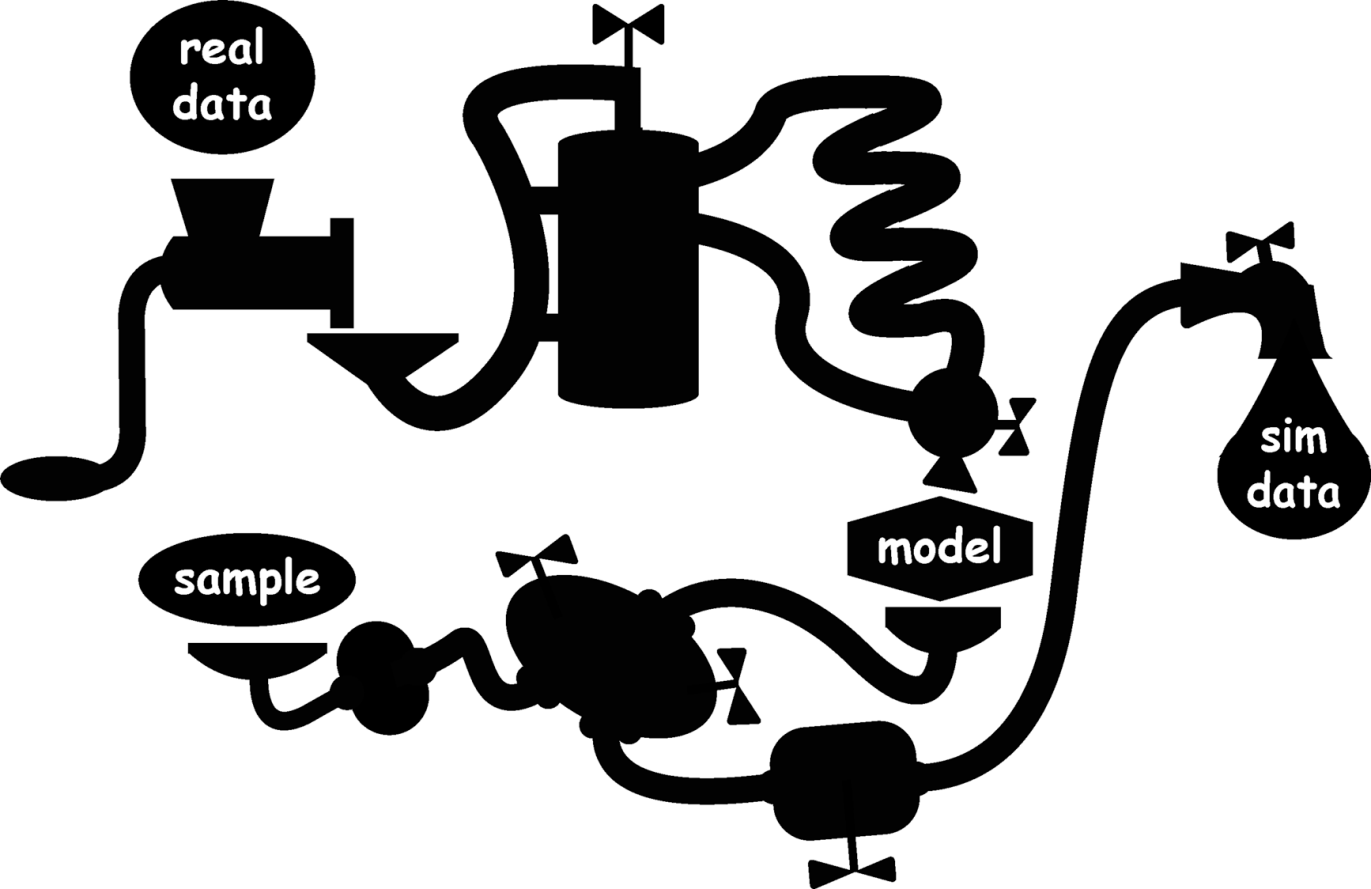

In recent years machine learning has made extensive progress in modeling many aspects of mass spectrometry data. We brought together proteomics data generators, repository managers, and machine learning experts in a workshop with the goals to evaluate and explore machine learning applications for realistic modeling of data from multidimensional mass spectrometry-based proteomics analysis of any sample or organism. Following this sample-to-data roadmap helped identify knowledge gaps and define needs. Being able to generate bespoke and realistic synthetic data has legitimate and important uses in system suitability, method development, and algorithm benchmarking, while also posing critical ethical questions. The interdisciplinary nature of the workshop informed discussions of what is currently possible and future opportunities and challenges. In the following perspective we summarize these discussions in the hope of conveying our excitement about the potential of machine learning in proteomics and to inspire future research.

## Introduction

Analytical workflows in proteomics frequently rely on analyzing proteins or peptides by liquid chromatography (LC) and tandem mass spectrometry (MS/MS). Machine learning has been applied to predicting peptide retention times and fragmentation spectra, but recent advances in deep learning have dramatically improved these predictions,^1−3^ as well as modeling other experimental steps, such as enzymatic digestion^4,5^ and ion mobility.^6^ Common to all steps is that the behavior of the proteins, peptides or ions can be predicted from amino acid sequences. However, no model exists that can make realistic predictions of data from a multidimensional separation and analysis of any sample or organism.

Machine learning is set to revolutionize the generation of realistic multidimensional data from arbitrary samples. There are several general application areas of such generative models. For example, when acquiring LC-MS/MS data, acquisition parameters are usually chosen from heuristics and past experience. A machine-learned model predicting LC-MS/MS data for diverse sample types in proteomics could replace guesswork and enable optimal experimental design, including for single cell analysis and other precious biological or clinical samples. Another application of such a generative model is that synthetic data could provide a benchmark for nearly any sample by creating synthetic data specific to sample processing, multidimensional separation, and data acquisition, regardless of whether the sample had been run before. Currently, system suitability and in-run quality control rely on measuring known samples to evaluate LC-MS/MS performance. If machine learning could be used to predict the expected results of different samples, gradients, and data acquisition, researchers would have a direct measure of the quality of any data set, independent of prior data from experimental reference samples. In turn, this would greatly improve the quality and reusability of nearly any acquired experimental data. Finally, generating an experimentally relevant synthetic ground-truth data set, i.e., known sequences, modifications, or differential abundances, will allow for benchmarking and identifying best performing search algorithms and statistical workflows.

These are three possible applications of such an integrated generative model, but there are countless areas of open scientific questions on future applications of machine learning in proteomics, ranging from data acquisition to biological interpretation. Although machine learning has been applied to individual phases of a proteomics experiment, efforts in combining these into one model have been limited, and there is no comprehensive model that can predict the data for any given sample type, sample preparation, or analysis method required for quality assessment, experimental optimization, or algorithm development. This is unsurprising as no individual researcher or research group has the necessary expertise in all aspects of a proteomics experiment. To foster collaboration between groups and across disciplines, including biology, biochemistry, analytical chemistry, physics, and computer science, we invited experts in these domains to a workshop on proteomics and machine learning held 14 to 18 March, 2022 in Leiden, The Netherlands.

**Figure 1:**
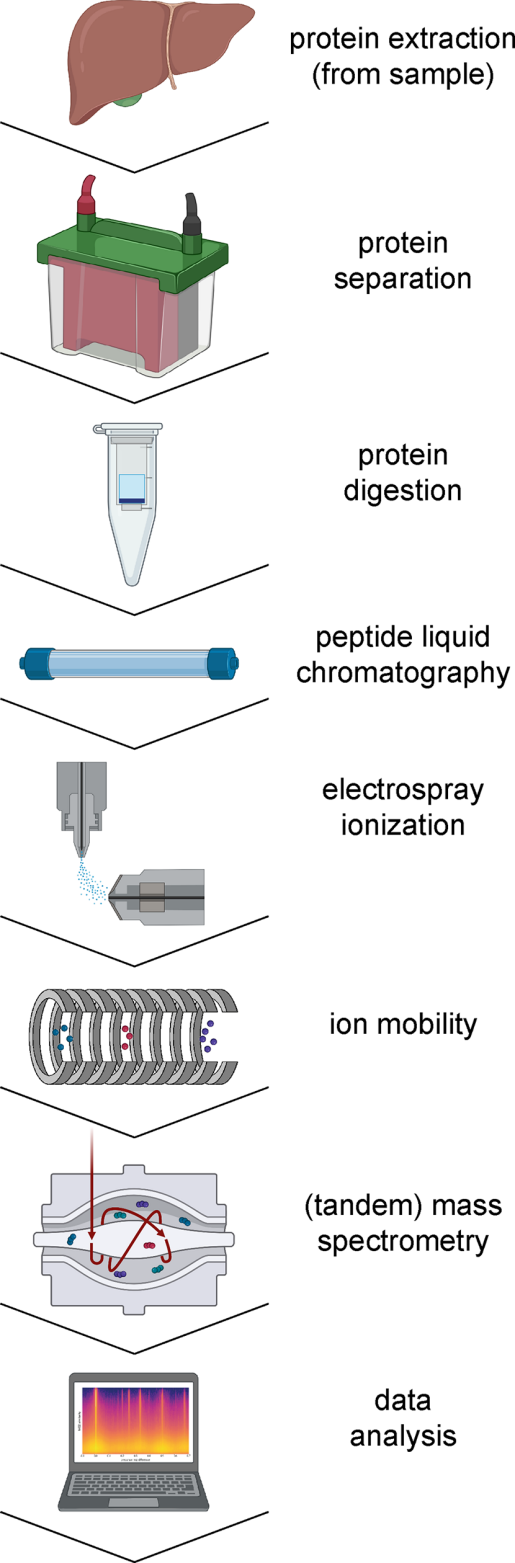
Some common steps in proteomics workflows corresponding to the workshop discussion topics and sections herein. Some icons made using BioRender.com.

The workshop was designed in early 2021 with an overall goal to discuss and deliver a conceptual design of an integrated machine-learned model of a proteomics experiment, covering all experimental steps from the sampling of the biological system to tandem mass spectrometry (Figure 1). This was accomplished by bringing together researchers who apply machine learning to individual steps of proteomics experiments, such as enzymatic digestion, chromatography, ion mobility, and tandem mass spectrometry, with experts on data repositories and open data formats, as well as practitioners of proteomics representing the end users of machine learned models. The general areas defined above provided a concrete framework for the discussions, including potential misuse of such models, i.e., in data fabrication, and possible social and engineering solutions to mitigate these risks. This summary of our workshop is not meant to be an exhaustive review of machine learning in proteomics, and specialties such as spatial proteomics or mass spectrometry imaging are not covered. We begin our summary of the workshop discussions by looking at trending topics in machine learning generally, and then ask how the proteomes themselves can be predicted.

## Trending Topics in Machine Learning

Machine learning, especially deep learning,^7,8^ has made remarkable progress in the past decade. Classically, machine learning could be roughly divided into supervised and unsupervised approaches, while current deep learning advancements have blurred these lines considerably and instead are easier to understand by delineating by use-case (i.e., generating data) and underlying model architecture (i.e., convolutional neural network). During the workshop, discussions were focused on current and future possibilities related to proteomic applications. This section consequently details some of the current machine learning trends that have the greatest potential for impact in proteomics.

Perhaps the most well-known machine learning application in modern day proteomics involves dimensionality reduction techniques such as t-SNE^9^ and UMAP,^10^ akin to classically defined unsupervised learning. Both approaches rely on an autoencoder architecture to reduce data noise and ambiguity of high-dimensional data. Notable uses of these approaches includes exploring relevant clusters in single cell proteomics data as well as inferring protein–protein interaction networks from high-dimensional combined RNA-Seq and proteomics data.^11^ In addition to dimensionality reduction techniques, multimodel representation learning^12^ is well suited for proteomics due to its ability to integrate features from different modalities and identify attributes that are shared and different. Successful applications, such as Contrastive Language–Image Pretraining (CLIP^13^), can integrate image and text data into a shared embedding by using a contrastive loss function that trains a model with pairs of positive and negative examples from the two modalities (image and text). Specific applications to mass spectrometry data include SpeCollate,^14^ GLEAMS,^15^ and yHydra,^16^ which are models that learn to jointly embed spectra and peptide sequence information into a shared latent representation. These techniques are pushing the boundaries of what is possible in data prediction and analysis in emerging proteomic techniques.

In addition to interrogating existing data, maybe the most interesting deep machine learning applications, and much of the focus of this paper, involve data generation. Generative approaches, such as generative adversarial networks (GANs^17^) and diffusion models,^18−20^ have shown impressive results mainly for image generation but also potential applications in data generation.^21,22^ Broadly, a GAN approach works by having a generator model play a game where it tries to fool its adversary, a classification model that tries to distinguish the fake data by the generator from genuine, real-world data. But training GAN networks are notoriously difficult because the competition between the two models can become unstable and training stops before reaching a satisfying optimum. Recently, GANs have been outpaced by a new class of generative models called diffusion or score-based models^18^ that learn a denoising function by applying it multiple times to a signal that is distorted on purpose, similar to denoising autoencoders. With ongoing optimization of speed and more resources, it is likely diffusion-based models will become ubiquitous for countless applications, including proteomics, in the coming years.

Until recently, each data type had a specific neural network layer architecture that it worked best with [i.e., convolutional neural networks (CNNs) were used mostly for images, while recurrent neural networks (RNNs) were used mostly for natural language text sequences], but a new universal layer architecture, the transformer, can work with any input and output data modality. Briefly, this is accomplished by using an attention-based architecture of stacked dense layers. First introduced in 2017,^23^ transformers have unified deep learning architectures to become the universal building block for various deep learning approaches. Many modern proteomics models already utilize transformers, including prediction of fragmentation spectra (e.g., Prosit Transformer^24^) or protein folding (e.g., AlphaFold^25^), and all of the approaches described above can or already use a transformer. Transformer-based models have captured the public’s attention with the ability to generate text (BERT,^26^ GPT-3^27^) or images (DALL-E-2^28^ and DreamStudio/Stable Diffusion^19^) from text prompts, and it is exciting to think of similar capabilities with respect to proteomic data generation. In addition to replicating sequential processes in a generative manner, transformers have also been used to successfully predict steps leading to an end point. Retrosynthesis^29^ is a relevant example, which seeks to determine reaction steps to reach a desired compound. Notably, state-of-the-art models often almost exclusively use transformers and do not rely on other concepts such as convolutional or recurrent layers.

While deep learning is currently the dominant technology in machine learning, it may be excessive for certain prediction tasks. There is a tendency to value solutions based on these complex algorithms currently in vogue more than simpler models that perform equally well, even if the simplest solution is recommended.^30^ There is likewise an issue of marginal gains when making very small, incremental improvements in prediction accuracy and/or sensitivity, without being able to show that these improvements have actual value in real life application, demonstrated by improved generalization and improved end result. The Benchmark Data Sets for Machine Learning in Proteomics section below describes tools and data sets that may help evaluate machine learning models. Finally, as these trending techniques are now applied in proteomics, new methods for machine learning are developed so rapidly that even the most visionary outlook on such topics is likely to quickly be outpaced.

## Proteomes and How to Predict Them

The ability to predict data from biology, and vice versa, is a primary focus of the topics discussed herein. Specifically, the ability to define the proteome of any species, sample type, and health status *a priori* is necessary for any downstream data prediction steps. Therefore, it is essential to reiterate concepts and highlight current resources of annotated genomes and tissue/biofluid/cell-specific proteome predictions (or empirical data). A proteome is broadly defined as the identity and abundance of proteoforms, which includes isoforms and post-translationally modified forms of each protein, in a given sample. The simplest representation of the protein sequences comprising the unmodified proteome may be derived from an annotated genome of a species, in humans encompassing approximately 20 000 canonical sequences.^31,32^ Recently, there is a greater appreciation for individual variation within a species, emphasizing the need for pan-genomes or study-specific genomes to accurately define the sequence space of the proteome. Beyond humans and common model systems such as mouse, zebrafish, and *Arabidopsis*, there is an explosion of genome annotations available from NCBI RefSeq,^33^ UniProtKB,^34^ and Ensembl,^35^ and third-party entities like DNA Zoo^36^ and the EarthBioGenome Project.^37^ Together, these resources provide the requisite search space for proteomic analysis in nearly any species or environmental sample.

Beyond the catalog of possible proteins in a species’ proteome, each tissue, biofluid, and cell-type contains a unique repertoire of proteins at varying abundance,^38,39^ which is also affected by health status (or organism level phenotype). Even so-called “housekeeping proteins” that are detectable in most cell types may vary in abundance.^38^ While there are various cell atlases and biofluid projects based on experimental proteomic data (Expression Atlas,^40,41^ Genotype-Tissue Expression (GTEx) proteome map,^42^ Human Plasma Proteome,^43^ Human Protein Atlas,^44^ Human Secretome,^45^ Immunological Proteome Resource,^46^ PeptideAtlas,^47^ and ProteomicsDB^48^), easily accessible lists of tissue/biofluid/cell-specific proteomes with abundance information are rare. Still, it is possible to reprocess proteomic data from public data sets, such as the Wang *et al.* 29 human tissue data set,^38^ Geiger *et al.* 28 mouse tissue data set,^39^ or the NCI Proteomics Data Commons cancer-centric data sets,^49^ to derive empirical lists of protein abundances by tissue/biofluid/cell-type and predict what proteins or proteoforms *may* be present in a given sample. Also, it is important to note that there are tissue/cell-specific databases of transcript abundance (such as ARCHS4,^50^ HPA Single Cell Type Atlas,^51^ and Tabula Sapiens^52^), which could be used as a proxy for protein abundance, albeit with the caveat that transcript and protein abundance is not directly correlated in tissues,^53^ while in biofluids transcript levels and secreted protein abundance therein are fundamentally disconnected since the source of the secretome is not cells in the biofluid itself.^45^ Although resources like these are largely focused on model organisms, with assumptions, caveats, and caution, tissue/biofluid/cell-type proteomes may be extrapolated between related species. Though all mammals are not primates or rodents, nor all plants are *Arabadopsis* or rice, this can provide an estimation of a proteome when prior knowledge is limited beyond an annotated genome of a related species.

Though these described resources provide confident predictions and thorough evidence of species- and tissue-specific proteomes, the proteomic data itself (i.e., instrumentally measurable signal) will not be a complete measurement of a predicted proteome, since protein extraction, protein digestion, peptide separation techniques, alternatively spliced isoforms, and post-translational modifications (PTMs) will affect the observability of the peptides and the inferred proteoforms. All of these hurdles may be overcome by combining annotated genomes and data repositories with current and future machine learning techniques. This will allow for accurate *in silico* predictions of proteomic data of a given sample type, identification of data from an unknown sample type, and describing experimental and technical artifacts in proteomic data.

A complex remaining task is the prediction of tissue- or organism level changes in protein abundance or post-translational modification, which are intrinsically linked to protein function. Machine learning may complement databases and ontologies to improve functional annotation of proteins, predicting function based on similarity to proteins with known functions in protein families,^54^ inferring function from coregulation of proteins found in large-scale proteomics studies^55^ or integrating protein and RNA-Seq data.^11^ Machine learning has already been used to predict functional relevance of phosphorylation by combining multiple databases and repositories.^56^ The proteome scale availability of three-dimensional protein structures from AlphaFold enables systematically investigating PTMs in their structural context to further improve our understanding of their functional relevance, and hence our ability to predict their changes.^57^

## Enzymatic Digestion

When students first learn about proteases or restriction enzymes, they are invariably told about their specificity as rules or discrete motifs. This is both a necessary and useful simplification. However, in practice, especially in proteomics, the reality is that not all cleavage sites are equal, and that proteolytic digestion is affected by residues outside the canonical motif, as well as PTMs and protein structure. Even if the proteins present in a sample are known, there is still a need to predict the resulting enzymatic peptides.

Predicting cleavage sites of proteins has already a long history, as B. Keil summarized in his analysis of tryptic cleavage in 1992,^58^ stating that trypsin would cleave after arginine (R) or lysine (K) except if it is followed by a proline (P), which is since known as the Keil rule. However, in the last two decades several groups have proven this assumption not always valid, as it has been found that the Keil rule is wrong in about 10% of all cases,^59^ that up to 40% of all tryptic peptides contain missed cleavages,^60^ that cleavage before proline is as common as cleavage before tryptophan, and even more frequent than cleavage before cysteine.^61^ Accurate prediction of cleavage sites has an impact on both types of proteomic analyses, identification, and quantification,^62^ though the impact on protein quantification is much higher. In identification, accurate cleavage prediction can reduce the search space and remove incorrect peptide candidates; however, in quantification, false cleavage estimations can strongly influence the calculated (relative) abundance of inferred proteins.^63,64^

Recent enzymatic prediction approaches make use of different machine learning techniques, such as random forests^62,65^ and deep learning.^4,5^ In all these approaches, various methods of training data curation are utilized and an amino acid window of size *n* around a known cleavage site is used as input for the learning algorithm. Most approaches, however, still rely on trypsin as a digestion enzyme, DeepDigest^5^ being one exception. Several other helpful tools have also been developed in the context of enzymatic digestion prediction, one of them being SPACEPro,^66^ a tool that analyzes cleavage efficiency using search results, which could potentially be used to curate training data for cleavage prediction algorithms.

With the recent success of models such as AlphaFold, which has been able to predict, at least to some accuracy, the structures of hundreds of millions of proteins,^25^ one could imagine including three-dimensional structure and kinetics as input features for machine learning algorithms to predict proteolytic cleavage on a given time scale, down to milliseconds,^67^ in different solvents,^68^ or even predicting structures of fragments and partially denatured proteins. Although attempts have been made in this direction already, there is still a need to develop more models for more enzymes, taking advantage of recent developments and factoring in tertiary structure and PTMs.

## Protein/Peptide Fractionation

Prediction of how proteins and peptides behave in electrophoretic or chromatographic separations is important in both analytical and preparative contexts, including when analyzing proteomics data from experimental workflows including multiple dimensions of separations or when optimizing preparative methods used to purify recombinant proteins, synthetic peptides, and other products.^69^ Such predictive models can also be used to optimize chromatographic fractionation in proteomics workflows, even if the optimization target is very different than when purifying a single component, for example adjusting chromatographic conditions to distribute the proteins or peptides evenly between fractions with minimal overlap.^70,71^

Electrophoresis has also been used both preparatively and analytically, and to fractionate proteomes for further analysis by liquid chromatography and mass spectrometry. The predicted electrophoretic migration can be compared to measurements using label-free quantitation, e.g., protein-level SDS-PAGE and peptide-level isoelectric focusing (IEF).^72^ Subsequently, this comparison can flag false positive identifications and covalent complexes (in SDS-PAGE). With increasing speed and sensitivity of mass spectrometers, there is now less perceived need for prefractionation methods in proteomics. However, IEF is still used in a variety of studies where additional separation or targeting of peptides is necessary. It is unknown to what extent machine learning would improve classical predictions, especially since the isoelectric point (pI) and molecular weight of a protein can be significantly affected by PTMs such as phosphorylation (affecting pI) or glycosylation (affecting both). Unfortunately, current pI calculators are not capable of predicting values for modified peptides. As IEF is still used for separation of modified peptides there is a potential to develop models that can predict the pI of modified peptides. For protein-level IEF under native conditions, a methodology similar to that recently used to calculate protein p*K*_a_ values^73^ using AlphaFold may be adopted.

In any continuous separation method, predicting distributions of analytes in discrete collected fractions is trivial given a prediction of the continuous retention or migration time, though special attention to peak widths and tailing may be warranted to accurately capture the overlap between fractions. A special case of protein and peptide separation methods is -the binary fractionation or enrichment of a subproteome or modification of interest, e.g., phosphorylation using titanium dioxide, immobilized metal affinity chromatography, affinity chromatography, or strong cation exchange, or glycosylation using hydrophilic interaction chromatography. Modern machine learning methods have not been applied to predict protein or peptide distribution in such fractionation approaches. Regardless, the direct benefits of accurately modeling protein and peptide behavior in chromatographic or electrophoretic fractionation methods warrants a need for such models, however crude, in our toolbox to enable data simulation of a wide range of proteomics experiments.

## Liquid Chromatography

Whereas the above dimensions of protein and peptide separation are increasingly used in special cases, such as enriching a part of the proteome, reversed-phase liquid chromatography is ubiquitously hyphenated with mass spectrometry in proteomics. Most commonly, the liquid chromatograph is physically coupled inline and online with the mass spectrometer through an electrospray interface, although fraction collection and off-line mass spectrometry via electrospray or MALDI is also possible. Online hyphenations treat time as a continuous variable, whereas in off-line methods time is discretized in intervals and typically not used in the further analyses. For a review on models and molecular simulation studies of reversed-phase liquid chromatography, see Lindsey et al.^74^

Models for retention time predictions have become increasingly complex since the seminal work by Meek published in 1980,^75^ driven by increasing availability and quality of training data with the advent of mass spectrometry-based proteomics. Peptide retention time predictions were first used to assist mass-spectrometry based identification in 2002.^76^ Artificial neural networks trained on amino acid compositions were introduced in 2003^77^ and later extended to take the actual sequence into account.^78^ Other contemporary models used sequence-derived features rather than raw sequences as input, such SSRCalc^79^ and ELUDE.^80^ More recently, deep neural networks have been trained on large, high-quality, data sets, resulting in even more accurate predictions.^2,81^ Recent implementations of these models have even been shown to even be able to predict previously unseen modifications.^3,82^

None of these methods attempt to predict elution profiles of peptides, though Afkham et al.^83^ compared experimental elution profiles with estimated uncertainties in retention time prediction in their GPTime model. Accurate modeling of elution profiles is critical in generating realistic synthetic data for optimizing proteomics experiments. Examples of the characteristics that should be modeled are the slope and length of the programmed gradient, the length of dynamic exclusion windows in data-dependent acquisition, and the window size/scan speed in data-independent experiments. Elution simulation here complements actual experiments and, when trained on data on the same chromatographic system and column, could be expected to produce very accurate predictions of retention times as well as chromatographic peak shapes. In targeted proteomics, elution profile prediction would be useful for minimizing interference when selecting peptides and transitions, as well as assist extracted ion chromatogram peak integration for robust quantitation. Elution profile predictions are also required to accurately model chimeric spectra (spectra containing product ions from multiple peptides) in synthetic LC-MS/MS data. These predicted chimeric spectra are useful for scoring identifications in the spectrum. Furthermore, data independent acquisition (DIA) specifically greatly benefits from knowing exactly what peptides contributed to a fragmentation spectrum.^84,85^

Chromatograms can be aligned using shared features, i.e., common peptides.^86,87^ Internal retention time standards^88^ have become popular in recent years. Although these add little information on the chromatographic separation to the thousands of peptides already in the sample, they simplify comparing data sets and automating data processing by allowing extrapolation to an indexed retention time (iRT) space. Accurate retention time and elution profile models can not only replace these internal standards for chromatographic alignment and system checks, but if the models are interpretable, they can also assist in troubleshooting, e.g., suggest if the mobile phase pH,^89^ temperature, or flow rate is wrong. Whether the goal is to optimize the chromatographic separation or other steps of a proteomics experiment, aligning chromatograms, or rescoring peptide-spectrum matches, realistic LC-MS/MS data simulation will benefit from realistic modeling of the chromatographic behavior of peptides, including elution profiles, and dependence on mobile phase and gradients. This also applies to modified peptides and other modes of chromatography that can be interfaced with mass spectrometry.

## Ion Mobility

Ion mobility is increasingly being used in proteomics as a fast separation or trapping method between the ion source and mass analyzer. Most, if not all, major mass spectrometry vendors now integrate ion mobility in their high-end instruments, although the technical implementations work along different principles (e.g., FAIMS, SLIM, TIMS, and TWIMS).^90^ The resolution of the different methods varies substantially,^91^ and higher resolution measurements are likely to require more complex algorithms to fit the data. These high-resolution ion mobility measurements are able to differentiate between isomeric structures^92^ that are unlikely to be correctly predicted by simpler models. This means that each ion mobility technique may require a different model, where simplicity while modeling most of the information should be preferred.

If used as a separation device, the ion mobility or arrival times can be calibrated into collisional cross sections (CCSs). Machine learning has already been used to predict CCS values for different classes of analytes^6,93,94^ and integrated in software for identifying unknown compounds.^92^ As both experimental resolution of ion mobility devices and accuracy of machine-learned models increase, the value of CCS prediction also increases. In cross-linking experiments, CCS values can be used to distinguish cross-linked peptides from unlinked peptides of similar mass-to-charge ratio.^95^ Comparing measured and predicted CCS values has also been used to interrogate protein structure and dynamics, including protein complexes.^96,97^

For some classes of molecules, CCS is highly correlated with the mass-to-charge ratio, limiting the usefulness of CCS prediction for the identification of unknowns or distinguishing between closely related species. Such lack of orthogonality should always be considered when evaluating the added value of applying machine learning in data analysis workflows. However, to generate realistic synthetic data from experiments including ion mobility separations requires at least some model of analyte behavior.

Ion mobility is becoming increasingly popular in bottom-up proteomics specifically. While the benefits to data acquisition are clear and significant by generating cleaner and more interpretable tandem mass spectra, the impact of predictions of ion mobility behavior or CCS values for peptide identification in untargeted experiments is currently limited, even if machine learned models are quite accurate.^6^ In large search spaces, such as variable PTMs, open modifications, or metaproteomics, CCS predictions could be valuable additions in analysis pipelines to reduce the number of possible candidates for peptide-spectrum matching. For the application of predicted CCS values in open searches, models should be developed that can accurately predict CCS for modified peptides.

## Mass Spectrometry

There are many choices in mass spectrometry-based proteomics. Top-down or bottom-up? With MS1 or MS2? Using CID or ETD? Low- or high-energy? Data-dependent or data-independent acquisition? Each combination is a different type of experiment, generating different information and requiring a different model to be trained.

The first stage of mass spectrometry, MS1, is acquired to provide accurate mass measurement of intact peptide ions, trigger data-dependent events, and generate quantitative information in experiments such as SILAC. Monoisotopic masses of intact peptide ions and the fragments thereof are easily calculated with sufficient precision based on atomic mass. However, monoisotopic mass is one of few properties of mass spectra that can be so easily calculated. Other isotopic peaks have contributions from multiple isotopologues, the relative abundance of which depend on sample origin, e.g., the fraction ^13^C in plants varies measurably with photosynthetic pathway.^98^ However, for most intents and purposes, isotopic distributions can be calculated from the elemental composition of the peptides and convolved by a theoretical or experimentally sampled peak shape that only depends on the mass-to-charge ratio.

The second stage, tandem mass spectrometry or MS2, is considerably more challenging to predict, and has been the topic of intense research going back at least to 1964 with the DENDRAL software for reconstructing molecular structures from fragment spectra.^99^ This was one of the first “expert systems” and therefore has a special place in the history of artificial intelligence. Tryptic peptide fragmentation patterns have been gradually refined from uniform predicted intensities for all b- and y-ions^100^ to separate intensities for b- and y-ions to intensities dependent on the neighboring residues. More recently, machine learning models such as MS^2^PIP,^1^ Prosit^2^ and others^101,102^ have been shown to produce even more accurate fragmentation pattern predictions. After calibrating the actual collision energy (versus instrument readback), Prosit is able to predict the intensities of b- and y-ions very close to experimental data.

Prediction of tandem mass spectra has many applications in proteomics. Accurate intensity predictions can replace flat intensities or simple models in peptide-spectrum matching algorithms. It is also possible to predict the peptide sequence directly from the spectra, rather than the other way around (i.e., *de novo* sequencing). Models that take collision energy into account can be used to optimize *in silico* collision energies for every targeted peptide in selected-reaction monitoring (SRM; and MRM/PRM), something which is extremely laborious to do experimentally. Simulation of tandem mass spectrometry of peptides is also a key component when simulating realistic proteomics data. This requires modeling the variability in fragmentation, which varies from peptide to peptide and depends on collision energies. In the future, models incorporating PTM prediction, coisolation, nontryptic, and semitryptic digestion, *etc.* will help identify possible peptides to clearly define and optimize model output.

## Peptide Observability and Proteotypicity

When combined, models for all experimental steps described above may be used to predict whether a peptide in a given sample will be detected by the mass spectrometer and “observed”. Different but related concepts and terms exist in this context, e.g., peptide observability or detectability, peptide quantifiability, or proteotypicity. There are different interpretations of these concepts, but key terms are defined as used in the context of this paper below. Peptide observability is the probability that a certain peptide can be identified in a certain sample given that the protein is present (at some level). Often, this probability is seen as binary classification, with the two classes of peptides termed “flyers” and “non-flyers”.^103^ Contrastingly, proteotypicity can be defined as the number of samples with a certain peptide divided by the number of samples with the protein containing that peptide.^2^

A peptide from a protein present in the biological sample may not be observable at all, for reasons such as poor protein extraction and solubilization, digestion efficiency, PTMs, peptide or protein degradation, suppression by coeluting peptides, stochastic sampling for fragmentation, and biases in the search algorithm. Even when a peptide is detected in a sample, it does not necessarily mean it is quantifiable.^104^

Modeling peptide observability requires modeling all the steps from the sample to the mass spectrometer, including peptide or protein fractionation, and enzymatic digestion. However, most proteomics data in repositories either lack these dimensions or the machine-readable metadata to use this information for machine learning, such as standardized and complete sample preparation protocols or the conditions and duration of proteolytic digestion. In general, a prediction model for peptide observability should be able to distinguish between a biological (i.e., nonrandom) missingness of a peptide and a technical (i.e., random) missingness. Proteins that are commonly seen in experiments where protein properties and sample preparation protocols are well-known and which yield an appropriate peptide distribution could be used as conditional input for such a peptide observability model. Ideal data sets for training models of peptide observability would be instrument-specific repetitive acquisitions from different institutes, different tissues, and different preprocessing methods, e.g., duration of proteolytic digestion. In addition, data sets of synthetic proteins including proteoforms and real-negative samples are helpful.

Several research groups have already addressed the problem of modeling peptide observability.^103,105−107^ Pino and co-workers created a model that combines observability with peptide ionization properties.^104^ In 2016, Edfors et al. determined gene-specific coefficients correlating mRNA and protein levels across 20 human tissues and cell lines as measured by RNA-Seq and SRM.^53^ Recently, Dincer et al. used a deep neural network model, Pepper, to derive sequence-specific coefficients describing the quantitative relationship (or bias) between the observed and measured peptide abundance, finding that the adjusted measurements correlate better than the unadjusted measurements with the RNA-Seq data in quantitative mass spectrometry data.^108^

To the best of our knowledge, no models currently predict the proteotypicity in samples enriched for subsets of peptides, e.g., phosphopeptides. Such prediction is challenging, in part due to the large experimental variability of the enrichment step. Prior knowledge of verified modification sites could be helpful in this case.

However, several applications can already take advantage of peptide observability prediction models. In intensity-based quantitation such as iBAQ,^109^ the number of observable peptides of a protein can be used to adjust the derived protein abundance. These methods would directly benefit from better estimates on peptide observability. The absence of peptides with high observability is more significant than the absence of those with low observability and may warrant further investigation. Conversely, and although unlikely to be identified as such, peptides with low predicted observability are likely problematic as candidate biomarkers, and high or consistent observability could be used to prioritize candidates.

In peptide identification by spectral libraries and database search engines, information on observability can be used as prior probabilities in peptide-spectrum matching, or to reduce the search space to peptides that are observable under the conditions of the experiment. During protein inference, proteins that cannot be unambiguously identified due to low observability of unique peptides can be excluded.

Although some work has already been performed in this context, there is still much work to be done. As always in machine learning, a variety of training data will be necessary to generate valid models, accompanied by proper, machine readable metadata, which is equally important. Efforts to collect appropriate training data sets has been ongoing for several decades (see Benchmark Data Sets for Machine Learning in Proteomics), and finally first steps have been taken to enhance the availability of the much-needed corresponding metadata.^110^

## Model Uncertainty

Typically, machine learning models in proteomics are evaluated on their accuracy rather than the uncertainty, error probability distributions, or confidence intervals that are important when incorporating the models in computational workflows.^111−114^ Software such as Triqler^115^ and MSeQUiP^116^ model error probability distributions for all steps from peptide-spectrum matching to protein quantification. This alleviates some of the systematic problems with sequential filters and as a consequence improves quantification of low abundance proteins. Other applications for modeling uncertainty can be found in peptide property prediction (e.g., retention time, ion mobility, charge, observability, and fragmentation or spectrum prediction). For retention time prediction, uncertainty estimates can improve the decision of which peptides to include during search.^83^ Modeling uncertainty of ion intensities is more complex as the combined intensity of one MS2 spectrum represents a joint fragmentation distribution for its isolated precursors. Interestingly, differentiating independent sources of uncertainty can be utilized to improve analyses. For example, there are methods that combine uncertainty estimates to align chromatograms and transfer identifications from one analysis to another.^117−119^ The same principle should be possible to apply to more complex predictions.

Bayesian techniques to model uncertainty in deep neural models include variational inference and Monte Carlo methods. Variational Inference methods learn the posterior distributions over the model’s weights, whereas Monte Carlo methods utilize random sampling instead. For example, Monte Carlo dropout^120^ makes several predictions for the same input while randomly ignoring a portion of the model’s weights. The resulting predictions are an approximation of the posterior distribution. Repeatedly sampling from different portions of a model is essentially mimicking an ensemble of models with a similar architecture. Such ensemble methods are their own theoretical framework to model uncertainty. Gaussian processes offer a framework for modeling uncertainty without the need for deep architectures. Particularly Gaussian process regression has been successfully used for estimating uncertainty of retention time predictions.^83^ A more comprehensive overview including other approaches can be found in the review by Abdar et al.^114^

Knowledge about the uncertainty of predictors could accelerate development of machine learning-driven proteomics. For instance, in training set generation, uncertainty estimates could be utilized to identify which subsets of data need more examples. During targeted proteomics assay development, these measures could be used to improve the exactness of peak prediction and retention time, thereby improving the assays. Confidence could also be transferred from spectral libraries to the peptide-centric analysis of DIA data. In general, computing confidence in single machine learning model predictions of LC-MS/MS peptide behavior, and generation of confidence intervals from peptide spectral matches (re)scoring models (e.g., using SVMs) will benefit protein identification and quantification.

## Simulating LC-MS/MS Data

Modeling arbitrary proteomics experiments requires models for all steps in Figure 1. Up to this point, the discussion has focused on what is needed to predict the observable peptides from any sample, and the last step is predicting tandem mass spectrometry data. Combined models can produce very realistic simulated data, which has numerous positive use cases, including experimental optimization, quality control using any sample, and benchmarking algorithms and bioinformatic workflows. Generating synthetic LC-MS/MS data in the mzML format is by no means novel. For example, the OpenMS infrastructure includes MSSimulator^121^ for simulating LC-MS/MS data and generating synthetic mzML files from FASTA files. More recently, the SMITER Python library^122^ and Synthedia^123^ have been used for simulating LC-MS/MS experiments and generate synthetic mzML files. The MaSS-Simulator^124^ and PhosFake (https://github.com/veitveit/PhosFake) have also been used to generate synthetic quantitative data or phosphopeptide features, respectively, in other file formats, for the explicit purpose of benchmarking algorithms.

However, recent developments in machine learning, such as GANs and diffusion models, and the specific efforts described above are likely to, when combined, generate far more realistic mzML files. As described herein, by taking into account predicted protein abundances, protein extraction, fractionation, digestion, chromatographic elution profiles of peptides, ionization, ion mobility, precursor selection and chimeric spectra, MS1 and MS2 spectra with all fragment ions, background, systematic and random mass measurement errors, and instrument drift, the synthetic data may be very close to data from real instruments. Such realistic, synthetic data would enable many new applications as discussed, but will also make it far easier to fabricate proteomics data that will be indistinguishable from real data. This inherent risk and possible mitigation were extensively discussed in a dedicated workshop session summarized in the following section.

Existing softwares for synthetic data generation, such as MSSimulator, are highly parametrized and include quite a few instrument aspects, making their realistic settings cumbersome at best. Still, these parameters could be trained using machine learning or dynamic programming methods to produce more realistic tandem mass spectra or any other output from an MS experiment. Software like MSSimulator and SMITER could easily be supplied with state-of-the-art (deep) learning models for predicting tandem mass spectra, e.g., Prosit or MS^2^PIP, as already done by Synthedia.^123^

One critical need resulting from this is that simulated LC-MS/MS data in the mzML format should be annotated as such, and the HUPO PSI controlled vocabulary, PSI-MS, extended with suitable terms to describe how the data was simulated, as opposed to generated by a real instrument, or at the very least that the mzML is simulated and the software or model used. Additional considerations for ensuring the integrity and veracity of mzML files are discussed in the section on Research Integrity.

A major new application of synthetic LC-MS/MS data is the *in silico* design and optimization of experiments. Instead of relying on instrument time or precious samples, methods and parameters can be simulated and optimized to yield the maximum amount of information possible, or sufficient information in the shortest possible time to answer a particular research question in an optimization of statistical power. Theoretically, this could reduce development time, improve the offerings in proteomics cores, and increase resulting data richness.

By simulating both samples and experimental configurations, researchers can also estimate what is reasonable data quality for a given sample, system, and method. This is a new dimension to quality control that does not compare data with a previously measured reference or standard but allows quality control metrics to be calculated on a single data set based on what is known about the data set, e.g., the organism, tissue, and experimental method, and comparing the resulting data with those predicted by machine learning from thousands or even millions of data sets from different organisms, tissues, and experiments. In other words, instead of relying on a specific standard or material to evaluate system suitability or experimental performance, any given sample can provide the same actionable information.

Lastly, realistic, synthetic LC-MS/MS data can be used to test, improve, and benchmark machine learning and classical algorithms and software. This can be accomplished by improving the output from database search engines using simulated spectra, generating ground-truth protein inference data to challenge protein inference algorithms, and generating ground-truth quantitative data to evaluate quantitative proteomics software. Unlike real ground-truth data, synthetic data sets would not have any systematic experimental errors from protein extraction or pipetting and could be created with the click of a button, instead of years of often tedious work preparing samples and curating spectra. Though it may seem that synthetic data may never “look” exactly like real data, it should be noted how advanced deep learning techniques have become at generating text and images that are nearly indistinguishable from those created by humans. We expect that crossing this uncanny valley is closer than it appears. Bridging it will create countless applications, including those listed above, and pose a formidable ethical dilemma.

## Research Integrity

Although the workshop focused on legitimate uses of realistic synthetic data, there is a possibility such data is passed off as real. This topic was discussed in detail, including mitigating social and engineering solutions. As most technologies, machine learning is amoral and can be used for beneficial or nefarious purposes alike. We hope that this pre-emptive discussion helps raise awareness and spur the development of safeguards.

Fraud is a problem of increasing concern in science, as various social and personal pressures entice individuals to make up data and attempt to publish it as legitimate research. In an extensive review of over 20 000 peer-reviewed publications containing images of Western blots, nearly 4% contained inappropriate manipulation.^125^ As this study was limited to Western blots, it only shows the “tip of the iceberg” of data fabrication in the biomedical literature. Unlike images, data fabrication in numerical tables can be subtle and hard to detect. In 2020, Bradshaw and Payne examined methods to detect fabricated numerical omics data,^126^ concluding that methods originally developed to detect fraud in banking and insurance would be applicable to scientific data.

There are already known examples of mass spectrometry data fabrication. In 2020, the University of Liverpool investigated a case of alleged research misconduct, finding falsification of key mass spectrometry data,^127,128^ including in the now-removed Figure 2 in a corrected paper.^127,129^ In this case, the misconduct was confirmed by extrinsic evidence such as equipment logs and financial records revealing the measurements were never performed. It would be naïve to think this is an isolated case, given the number of Western blots found to be manipulated, when these were systematically investigated.

The workshop session focused on data fabrication and falsification using generative machine learning.^22^ Data fabrication could happen at different stages of proteomics experiments: experimental data acquisition, manipulation of existing raw data, and downstream data analyses. We will not here provide details on how this can be done, but the workshop demonstrated that it is currently possible to fabricate proteomics data in ways that are not all trivial to detect. However, there are also several engineering and social solutions to mitigate risks of realistically simulated data being passed off as real.

Manipulated or fabricated data can be detected by a variety of algorithms leveraging number theory metrics unrelated to study design, such as Benford’s Law^130^ used by Bradshaw and Payne,^126^ and Zipf’s Law.^131^ The workshop demonstrated that some existing models for predicting mass spectra violate some of these laws. Beyond number theory, detection algorithms can be strengthened by domain knowledge and look for expected patterns in different data layers and comparing measured masses, peak shapes and isotopic distributions with the limits imposed by the instrument settings. It is possible to calculate these metrics from public data in repositories to obtain the limits and expected values of these metrics, assuming that this data is in fact real. One can also check for expected correlations in quantitative data, based protein-peptide stoichiometry or coexpression. If data is generated by spiking synthesized peptides of interest into the sample, and then measuring it using an actual mass spectrometer, one may be able to find remnants of the peptide synthesis in the data.

In addition to algorithmic fraud detection, it would be advantageous for the community to support a limited set of tools that generate synthetic data, which are built with data integrity in mind. There are at least two important features of such tools. First, they should target the open standard mzML file format, rather than vendor formats. The latter could be made more immutable by encoding timestamps, location and instrument serial numbers, file hashes, and theoretically even using technologies such as blockchain that prove a particular data set was acquired on a particular instrument in a particular location at a particular time. One drawback is that it then becomes impossible to fully anonymize data. Data anonymization may be preferred when publishing certain raw data, for example from interlaboratory comparisons or ring trials. Fortunately, with many vendors moving into clinical trials, many of their formats are already relatively immutable.

A second safeguard are digital watermarks in synthetic data. This can be accomplished by inserting patterns in the signal noise or features that do not match any known protein sequence (e.g., “FAKEPEPTIDE” amino acid sequence). These watermarks should be cumbersome to remove but not interfere with any data analysis. This safeguard requires that at least some components of the generating software are not provided as open source. As with security features in banknotes, some watermarks can be publicly known and easy to detect, while others would be confidently shared with journals and repositories, and some known only by the tool developers. Overall, this would allow synthetic data to be used without risking them being mistaken for real.

Beyond engineering solutions, there are also necessary social solutions. Given that data fabrication or duplication can happen at different levels, it is crucial to have access to the data provenance from raw data to reported results, requiring that all data, including raw data in closed vendor formats, and code are made available before publication. Such solutions already exist in the form of the ProteomeXchange repositories^132^ and ability to provide data analysis scripts or notebooks as Supporting Information or on a public server such as OSF (https://osf.io/) or GitHub (https://github.com), containing all steps from the raw data in the repository to the figures and tables in the publication. Ensuring this information is actually provided requires social solutions such as journal policies requiring raw data in vendor formats to be deposited and educating reviewers and editors about these topics.

In summary, it is important the proteomics community is aware of the increasing ease with which realistic synthetic proteomics data can be generated using machine learning. The prevalence of problematic Western blots in the literature should be a cause for reflection, as these are far cheaper to perform or repeat than state-of-the-art proteomics experiments requiring million-dollar instruments and reagents for thousands of dollars. At the same time, there are many legitimate use cases for simulating proteomics data, as outlined in the Introduction, and with a combination of engineering and social solutions, the risks that such realistic simulated data is being passed off as real undetected can be reduced.

## Benchmark Data Sets for Machine Learning in Proteomics

The workshop discussed the steps needed to develop a unified model to generate synthetic proteomic data, discussed possible applications of this capability, and touched on ethical concerns therein. However, to effectively catalyze this research topic, suitable proteomics data sets must be accessible to machine learning practitioners, as well as machine learning methods to data generators. Accessible and fit-for-purpose benchmark data sets are essential in domains such as machine learning for education and calibration across the field. For instance, the infamous Anderson’s *Iris* data set^133^ continues to be used in most beginner tutorials to demonstrate supervised and unsupervised machine learning based on petal and sepal length and width. More advanced machine learning modelers typically move on to the Titanic data set^134^ with more attributes and larger sample size. These two example data sets are accessible to newcomers and experts alike because of their small size, fit for purpose due to their straightforward attributes, and their frequent use. Together this results in an abundance of available tutorials across different software, languages, and algorithms, all based on the same underlying data set. Having benchmark data sets like these creates a point of entry with a low threshold, easy for beginners and education, but more importantly, these data sets can be used to explore and benchmark current and new machine learning techniques, irrespective of domain knowledge of the data set itself.

Specific to proteomics applications of machine learning, there is a need for similarly accessible and fit for purpose benchmark data sets. Although many machine learning endeavors such as DeepLC^3^ and Prosit^2^ validate against external data sets from laboratories that were not used during model training, the community has not agreed on specific benchmark data sets. Such benchmark data sets would make comparisons of different models more informative. Many of these deep learning models are also trained on samples from the same data sets, for example the data sets of synthetic peptides from ProteomeTools,^135^ and while these are a great resource for homogenized training, it can be hard to compare across tools without any collectively acknowledged inference source. Additionally, tools have recently been released that make it easier for researchers to extract machine learning-ready data from raw files, such as MS2AI,^136^ but even this is limited due to the complexity and variety of instruments and software used in proteomics.

In the months following the workshop, participants developed a resource to address this need.^137^ProteomicsML.org provides an online repository of easily ingested data sets with attributes spanning peptide properties and mass spectrometry data types, as well as companion tutorials on training deep learning models. This dynamic resource of proteomics benchmark data sets will be curated similar to the UCI Machine Learning Repository^138^ or MoleculeNet,^139^ and is mirrored on a GitHub repository to enable programmatic access (https://github.com/ProteomicsML/ProteomicsML). The benchmark data sets in ProteomicsML.org are optimally sized data matrices, with data import ease and handling as the goal. The specific search settings and filtering used to create each data set is described so that users can also reprocess from source raw mass spectrometry files (as PRIDE^140^ or MassIVE^141^ identifiers), but importantly, this is not required for using the data sets in machine learning applications. As described in prior sections, there are numerous machine learning applications in proteomics, and the data sets at ProteomicsML.org are also organized by application (i.e., retention time, spectrum prediction, ion mobility cross-section, enzymatic digestion, peptide observability/proteotypic peptides, peptide fractionation). The ProteomicsML.org resource will grow with community involvement, including both training and testing data sets by application, and larger data sets to expand alongside computational and instrument advances, helping machine learning experts experiment with proteomics data, and proteomics experts learn about machine learning applications.

## Conclusions

Taken together, the concepts and issues presented here provide a relevant, and hopefully exciting overview of the status of machine learning in proteomics today, and a few possible paths forward. It is clear that machine learning in proteomics has not just exploded in recent years, but is in fact here to stay. The breakthroughs in peptide identification performance alone have been quite impressive, and still new work continues to improve upon the previous state-of-the-art.

Perhaps most notably, the individual models that have been built so far could be assembled into a larger, end-to-end model, which could then predict realistic, synthetic data, which in turn could have many positive uses, as outlined in detail above, but that could also pass for real data if a nefarious person offered such synthetic data up as actual acquired data. Appreciating this capability, albeit theoretical, creates novel issues in proteomics that are deserving of attention. Concrete steps can be taken to safeguard against the potential issues raised by realistic synthetic data, while ensuring that positive uses may be pursued without issue. Rather than wait for the problem to occur (or be noticed), it would be prudent if the field began preemptively setting up appropriate safeguards, especially concerning the low-hanging fruit of adapting standard mass spectrometry file formats to accommodate well-annotated and clearly flagged synthetic data, while also ensuring that instrument raw files are fingerprinted or marked in a way that would allow their provenance to be traced to the actual instrument if needed. The latter comes with its own issues regarding data generator anonymity, and should therefore likely be engineered in such a way as to only be possible upon specific request.

However, before building such comprehensive and believable mass spectrometry data generators, more sophisticated models will likely be necessary. These models in turn will require ever better training data and adequate benchmarking data. Here too, there are several key issues to address moving forward, notably with regard to metadata provisioning. Currently, metadata provisioning in proteomics is scarce at best, and can frequently be found to be incorrect upon *a posteriori* verification.^142^ A more diligent annotation of data deposited in the public domain would provide much-needed leverage for a variety of downstream uses, which include building better machine learning models.

The power of machine learning as a nearly universal tool has created a modern-day Maslow’s (jack)hammer,^143^ and likewise machine learning is not always the best tool for the job. Many prediction tasks in proteomics do not require machine learning. As a trivial example, theoretical fragment ion masses can be easily calculated by summing the monoisotopic masses of the corresponding amino acid residues and applying the ion-specific mass loss correction. Though machine learning is making bold strides in modeling nearly every aspect of a mass spectrometry-based proteomics experiment from the biological system to interpretation of the results, the gains offered by machine learning should be critically evaluated. The curated data sets of ProteomicsML.org are useful for both beginners and experienced practitioners of machine learning, and for benchmarking and quantifying the gains of new models. With the accelerating growth in size and complexity of proteomics data, machine learning will become increasingly indispensable and fundamentally change the way proteomic data is acquired and interpreted.

## Abbreviations

ARCHS4: all RNA-seq and ChIP-seq sample and signature search
BERT: Bidirectional Encoder Representations from Transformers
CCS: collisional cross sections
CID: collision-induced dissociation
CLIP: Contrastive Language–Image Pretraining
DALL-E-2: Machine learned model for generating images from text descriptions (portmanteau of WALL-E and Dali)
DENDRAL: Dendritic Algorithm
DIA: data independent acquisition
ELUDE: unknown; wordplay on elution seems likely
Ensembl: just a name
ETD: electron-transfer dissociation
FAIMS: high field asymmetric waveform ion mobility spectrometry
FASTA: derived from the FASTP (P for protein) similarity search, FASTA is short for Fast All, and the file format used became known as fasta.
GANs: generative adversarial networks
GLEAMS: a learned embedding for annotating mass spectra
GPT-3: Generative Pretrained Transformer 3
GPTime: Gaussian processes time
HPA: Human Protein Atlas
HUPO PSI: Human Proteome Organization Proteomics Standards Initiative
iBAQ: intensity-based absolute quantification
IEF: isoelectric focusing
iRT: indexed retention time
LC: liquid chromatography
MALDI: matrix-assisted laser desorption/ionization
MassIVE: mass spectrometry interactive virtual environment
MaSS-Simulator: made to differentiate from similar tools: MSSimulator and MS-Simulator
MRM: multiple reaction monitoring
mRNA: messenger ribonucleic acid
MS/MS: tandem mass spectrometry
MS2A: mass spec to artificial intelligence
MS^2^PIP: MS2 peak intensity prediction
MSeQUiP: method for quantifying uncertainty in peptide-spectrum matches
MuSIC: multiscale integrated cell
mzML: official HUPO-PSI standard format for mass spectrometry data, derivative of previous mzXML format, mz (mass-to-charge ratio) and XML (eXtensible Markup Language)
NCBI RefSeq: National Center for Biotechnology Information Reference Sequence Database
NCI: National Cancer Institute
OSF: Open Science Framework
pI: negative log (base 10) of isoelectric point
p*K*_a_: negative log (base 10) of acid dissociation constant
PRIDE: Proteomics Identification Database
PRM: parallel reaction monitoring
ProteomicsDB: DB is short for database
PTMs: post-translational modifications
RNA-Seq: ribonucleic acid sequencing
SDS-PAGE: sodium dodecyl sulfate-polyacrylamide gel electrophoresis
SILAC: stable isotope labeling by/with amino acids in cell culture
SLIM: structures for lossless ion manipulation
SMITER: Synthetic mzML writer
SPACEPro: Shotgun Proteomic Analysis of Cleavage Efficiency of Proteins
SRM: selected-reaction monitoring
SSRCalc: sequence-specific retention calculator
SUMOylation: small ubiquitin-like modifier modification
SVM: support vector machine
TIMS: trapped ion mobility spectrometry
t-SNE: t-distributed stochastic neighbor embedding
TWIMS: traveling wave ion mobility spectrometry
UCI: University of California, Irvine
UMAP: Uniform Manifold Approximation and Projection
UniProtKB: Universal Protein Resource Knowledgebase
